# Draft Genome Sequence of *Picocystis* sp. Strain ML, Cultivated from Mono Lake, California

**DOI:** 10.1128/MRA.01353-18

**Published:** 2019-01-24

**Authors:** Emily N. Junkins, Blake W. Stamps, Frank A. Corsetti, Ronald S. Oremland, John R. Spear, Bradley S. Stevenson

**Affiliations:** a Department of Microbiology and Plant Biology, University of Oklahoma, Norman, Oklahoma, USA; b Department of Civil Engineering, Colorado School of Mines, Golden, Colorado, USA; c Department of Earth Sciences, University of Southern California, Los Angeles, California, USA; d U.S. Geological Survey, Reston, Virginia, USA; University of Maryland School of Medicine

## Abstract

The microscopic alga Picocystis sp. strain ML is responsible for recurrent algal blooms in Mono Lake, CA. This organism was characterized by only very little molecular data, despite its prominence as a primary producer in saline environments. Here, we report the draft genome sequence for Picocystis sp. strain ML based on long-read sequencing.

## Abstract (precis)

The microscopic alga Picocystis sp. strain ML is responsible for recurrent algal blooms in Mono Lake, CA. This organism was characterized by only very little molecular data, despite its prominence as a primary producer in saline environments.

## ANNOUNCEMENT

Mono Lake, a hypersaline soda lake in eastern California, sustains a population of the oxygenic alga Picocystis sp. strain ML. Previous studies have described this strain, a close relative to Picocystis salinarum (1), as a major primary producer in the lake (2). Members of the genus Picocystis have also been cultivated from East Africa (3, 4), Inner Mongolia, China (5), and, more recently, Peru (6). Despite its role as a primary producer and its global distribution, nothing is known of its genomic potential. A recent study characterized a bloom using metagenomic and transcriptomic approaches and suggested that Picocystis sp. strain ML produced photosynthetic transcripts, potentially producing oxygen, at low-light depths (7). Here, we report the draft genome sequence of Picocystis sp. strain ML, which was previously estimated to be 23 Mbp (8).

**FIG 1:**
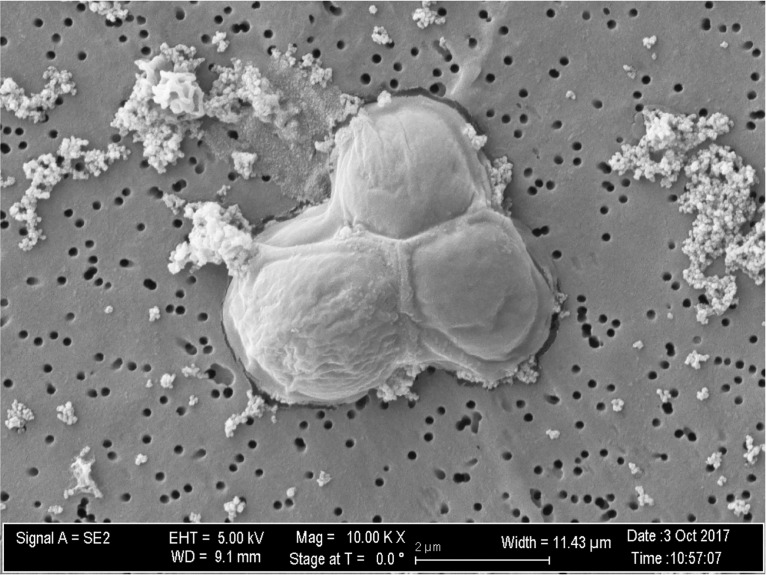
Scanning electron microscopy (SEM) image of *Picocystis* sp. strain ML on a polycarbonate filter. Scale bar = 2 µm.

A sample collected from 20-m depth in Mono Lake (7) was inoculated into L1 liquid medium (product number MKL150L; National Center for Marine Algae). Upon visualization of growth, a sample was spread onto L1 agar (1.0% [wt/vol]) for isolation, and a single colony was used to inoculate L1 medium for DNA extraction. Axenic status was determined by a lack of growth in marine purity broth (9) and using scanning electron microscopy (SEM) (Fig. 1). Volumes of 250 µl were passed through 0.1-µm polycarbonate filters, and the retained cells were fixed (0.75% ruthenium red, 50% glutaraldehyde, 1 M HEPES) and sputter coated (5 nm AuPd) with a Hummer V1 sputtering system (Anatech USA). Samples were viewed for axenic status on a Zeiss NEON field emission gun-SEM (FEG-SEM) dual-beam high-resolution system with an energy selective backscatter (EsB) detector (Zeiss). High-molecular-weight DNA was extracted via a modified cetyltrimethylammonium bromide (CTAB) extraction, purified with Sera-Mag SpeedBeads (GE) via the AMPure XP protocol (Agencourt), and quantified using a Qubit fluorometer (final concentration, 166 µg/ml). A genomic library was prepared using the PacBio SMRTbell template prep kit 1.0-SPv3 (Pacific Biosciences). The final library was size selected at 10 kb (Blue Pippin; Sage Science) and sequenced on a PacBio Sequel (Pacific Biosciences) using 4 single-molecule real-time (SMRT) cells via 2.0 chemistry, with a 10-h movie. To remove any contaminating bacterial sequences, reads were filtered with custom scripts for those that taxonomically matched the phylum Chlorophyta based on Kaiju version 1.6.2 classification (10). After quality control, a total of 251,086 reads were assembled using Canu version 1.6, generating 318 contigs, with a coverage of 40.77×, a total assembly length of 29.6 Mbp, a GC content of 53.6%, and an *N*_50_ value of 154 kbp (11).

A total of 14 small subunit (SSU) rRNA regions were found using RNAmmer version 1.2, closely matching (99.7% ± 0.67%) that of P. salinarum L7 (GenBank accession number AF153313) or matching (99%) the chloroplast of P. salinarum CCMP:1897 (GenBank accession number KJ746599) based on BLAST alignment (12, 13). RepeatMasker version 4.0.7 was used to mask repetitive elements (0.11%) with RMBlast version 2.6.0 (14) before gene prediction using AUGUSTUS trained to Chlamydomonas reinhardtii, the most closely related green algal model (15). A total of 5,613 coding regions were detected, of which 40.4% were characterized using BLASTKoala version 2.1 (16). The final assembly represents the first publicly available draft genome sequence of Picocystis sp. strain ML.

During the recent bloom, no genes were observed for ammonium oxidation from ammonia-oxidizing bacteria (AOB), unlike in previous years (17), and it was speculated that Picocystis sp. strain ML was assimilating these compounds (7). This genome revealed the presence of solute carrier family (commonly referred to as SLC) ammonium transporters, illustrating the genetic potential for Picocystis sp. strain ML to take in ammonium ions, perhaps explaining the lack of ammonium oxidation transcripts in the water column and the lack of AOB in such a monoculture environment. Overall, this genome sheds light on a primary producer’s genetic potential in a unique aquatic ecosystem.

### Data availability.

This whole-genome shotgun project has been deposited in DDBJ/ENA/GenBank under the accession number QYZS00000000. The version described in this paper is version QYZS01000000. Raw sequence reads have been deposited in the SRA database under BioProject number PRJNA490491. Custom scripts and software settings are available on GitHub at https://github.com/emilyjunkins/PicoML/tree/v1.0 and https://doi.org/10.5281/zenodo.2366252.
